# From SARS to MERS, Thrusting Coronaviruses into the Spotlight

**DOI:** 10.3390/v11010059

**Published:** 2019-01-14

**Authors:** Zhiqi Song, Yanfeng Xu, Linlin Bao, Ling Zhang, Pin Yu, Yajin Qu, Hua Zhu, Wenjie Zhao, Yunlin Han, Chuan Qin

**Affiliations:** 1 Institute of Laboratory Animal Science, Chinese Academy of Medical Sciences (CAMS) & Comparative Medicine Centre, Peking Union Medical Collage (PUMC), Beijing 100021, China; songzhiqi1989@foxmail.com (Z.S.); xuyanf2009@163.com (Y.X.); bllmsl@aliyun.com (L.B.); zhangling@cnilas.org (L.Z.); pinyucau@gmail.com (P.Y.); quyj@cnilas.org (Y.Q.); zhuh@cnilas.org (H.Z.); hnndwenjiezhao@163.com (W.Z.); 18510165683@163.com (Y.H.); 2 NHC Key Laboratory of Human Disease Comparative Medicine, the Institute of Laboratory Animal Sciences, CAMS&PUMC, Beijing 100021, China; 3 Beijing Key Laboratory for Animal Models of Emerging and Reemerging Infectious, Beijing 100021, China

**Keywords:** coronaviruses, SARS-CoV, MERS-CoV, spike proteins, animal model, prevention and treatment

## Abstract

Coronaviruses (CoVs) have formerly been regarded as relatively harmless respiratory pathogens to humans. However, two outbreaks of severe respiratory tract infection, caused by the severe acute respiratory syndrome coronavirus (SARS-CoV) and the Middle East respiratory syndrome coronavirus (MERS-CoV), as a result of zoonotic CoVs crossing the species barrier, caused high pathogenicity and mortality rates in human populations. This brought CoVs global attention and highlighted the importance of controlling infectious pathogens at international borders. In this review, we focus on our current understanding of the epidemiology, pathogenesis, prevention, and treatment of SARS-CoV and MERS-CoV, as well as provides details on the pivotal structure and function of the spike proteins (S proteins) on the surface of each of these viruses. For building up more suitable animal models, we compare the current animal models recapitulating pathogenesis and summarize the potential role of host receptors contributing to diverse host affinity in various species. We outline the research still needed to fully elucidate the pathogenic mechanism of these viruses, to construct reproducible animal models, and ultimately develop countermeasures to conquer not only SARS-CoV and MERS-CoV, but also these emerging coronaviral diseases.

## 1. Introduction

Before the first outbreak of severe acute respiratory syndrome (SARS), a limited number of coronaviruses were known to be circulating in humans, causing only mild illnesses, such as the common cold [1]. Following the 2003 SARS pandemic [2,3], it became apparent that coronaviruses could cross the species barrier and cause life-threatening infections in humans. Therefore, further attention needs to be paid to these new coronaviruses.

The 21st century has seen the worldwide spread of two previously unrecognized coronaviruses, the severe acute respiratory syndrome coronavirus (SARS-CoV) [4] and Middle East respiratory syndrome coronavirus (MERS-CoV), both of which are highly pathogenic. Starting from November 2002 in China [5], there have been unprecedented nosocomial transmissions from person to person of SARS-CoV, accompanied by high fatality rates. A united global effort led to the rapid identification of the SARS coronavirus and remarkable scientific advancements in epidemic prevention. Additionally, the zoonotic transmission of SARS from December 2003 to January 2004 [6] provided insight for researchers into the origin of this novel coronavirus. Notably, the SARS pandemic was declared to be over in 2004 when no more infections in patients were being detected. Subsequently, certain SARS-CoV-like viruses found in bats demonstrated the ability to infect human cells without prior adaptation [7,8] which indicates the possibility of the re-emergence of SARS-CoV or SARS-CoV-like viruses.

A decade later in June 2012, another highly pathogenic and novel coronavirus, MERS-CoV, was isolated from the sputum of a male patient who died from acute pneumonia and renal failure in Saudi Arabia [9]. Nosocomial infections were reported, and international travel led to the transmission of MERS-CoV to countries outside of the Arabian Peninsula, causing it to become a global pathophoresis. In May 2015, an outbreak of MERS occurred in South Korea due to an individual returning from the Middle East [10]. Based on the lessons learned from managing SARS-CoV prevalence over the last decade, tremendous progress toward unraveling the biological characteristics of MERS-CoV has been achieved at an unprecedented speed. Scientific advancements have allowed for rapid and systemic progress in our understanding of the epidemiology and pathogenesis of MERS-CoV.

SARS-CoV and MERS-CoV share several important common features that contribute to nosocomial transmission, preferential viral replication in the lower respiratory tract, and viral immunopathology. This review highlights the epidemiology and pathogenesis of these viruses, including our current understanding of their biological characteristics, their transmission, and their replication in the host. The spike proteins (S proteins) of CoVs play pivotal roles in viral infection and pathogenesis. As critical surface-located trimeric glycoproteins of CoVs, they guide entry into host cells. In this review, we summarize the structure and function of the S proteins and therapeutics designed to target them. Moreover, we will explore how CoV–host interactions cause pathogenic outcomes and discuss potential treatment options, as well as describe recent mammalian models that closely recapitulate the pathogenic process and have contributed to the development of prevention and treatment strategies for SARS-CoV and MERS-CoV. Although several potential therapies have been identified with SARS and MERS in animal and in vitro models, human clinical trials remain lacking, hindering the advancement of these potential countermeasures.

## 2. Epidemiology of SARS-CoV and MERS-CoV

Prior to the outbreaks of SARS and MERS [2,9], the clinical importance and epidemic possibility of CoVs had been recognized by researchers, (Table 1). In 2002, a SARS epidemic that originated in Guangdong Province in China resulted in 916 deaths among more than 8098 patients in 29 countries [11], identifying SARS as the first new infectious disease of the 21st century. Ten years later, the World Health Organization (WHO) published 2254 laboratory-confirmed cases of MERS-CoV that occurred from 2012 to 16 September 2018, with at least 800 deaths in 27 countries. Remarkably, more than 80% of recent research into the virology and genetics of this infection indicated that bats could be the possible natural reservoirs of both SARS and MERS-CoV. Palm civets [12] and dromedary camels [13] are also possible intermediary hosts of SARS and MERS, respectively, before dissemination to humans [14].

The transmission mechanism of SARS-CoV and MERS-CoV has yet to be fully understood. For transmission from animals to humans, direct contact with the intermediary host might be one route. Recent reports demonstrated that camel workers in Saudi Arabia with high prevalence of MERS-CoV infection may contribute to the transmission of MERS [15]. Some customs and habits may also be conducive to transmission, such as the consumption of milk, urine, or uncooked meat. In this way, MERS-CoV was transmitted from dromedary camels directly to humans, principally in the Arabian Peninsula, and this is considered to be the main route of transmission from animals to humans, causing significant morbidity and mortality [16,17]. Human-to-human spread has also been detected, especially through nosocomial transmission. Delays in diagnosis in hospitals might lead to secondary cases among healthcare workers, family members, or other patients sharing rooms [18,19,20,21,22]. Among the reported cases of SARS, 22% were healthcare workers in China and more than 40% were healthcare workers in Canada [23]. Nosocomial transmission for MERS has similarly been seen in the Middle East [16] and in the Republic of Korea [22]. Outbreaks in other countries all resulted from the reported cases in the Middle East or North Africa, and transmission was the result of international travel. Both SARS and MERS caused large outbreaks with significant public health and economic consequences.

## 3. Pathogenesis of SARS-CoV and MERS-CoV

Although our current understanding of the pathogenesis of the SARS-CoV and MERS-CoV infection remains unclear, we summarize what is presently known (Table 2).

**Figure 1:**
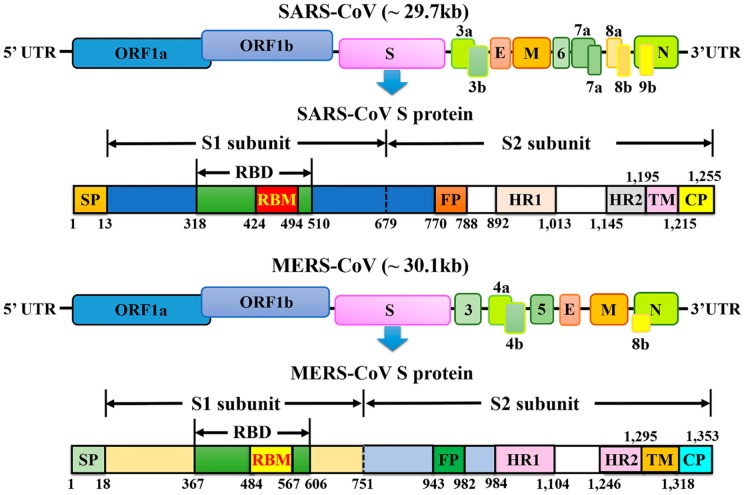
Schematic representation of the genome organization and functional domains of S protein for SARS-CoV and MERS-CoV. The single-stranded RNA genomes of SARS-CoV and MERS-CoV encode two large genes, the ORF1a and ORF1b genes, which encode 16 non-structural proteins (nsp1–nsp16) that are highly conserved throughout coronaviruses. The structural genes encode the structural proteins, spike (S), envelope (E), membrane (M), and nucleocapsid (N), which are common features to all coronaviruses. The accessory genes (shades of green) are unique to different coronaviruses in terms of number, genomic organization, sequence, and function. The structure of each S protein is shown beneath the genome organization. The S protein mainly contains the S1 and S2 subunits. The residue numbers in each region represent their positions in the S protein of SARS and MERS, respectively. The S1/S2 cleavage sites are highlighted by dotted lines. SARS-CoV, severe acute respiratory syndrome coronavirus; MERS-CoV, Middle East respiratory syndrome coronavirus; CP, cytoplasm domain; FP, fusion peptide; HR, heptad repeat; RBD, receptor-binding domain; RBM, receptor-binding motif; SP, signal peptide; TM, transmembrane domain.

Coronaviruses are the largest kind of positive-strand RNA viruses (26–32 kb) as they are about 125 nm in diameter [24], and comprise four genera (alpha-, beta-, gamma-, and delta-coronavirus) [25]. Currently, six human CoVs (HCoVs) have been confirmed: HCoV-NL63 and HCoV-229E, which belong to the alpha-coronavirus genus; and HCoV-OC43, HCoV-HKU1, SARS-CoV, and MERS-CoV, which belong to the beta-coronavirus genus. SARS-CoV and MERS-CoV are the two major causes of severe pneumonia in humans and share some common coronavirus structural characteristics. Similarly, their genomic organization is typical of coronaviruses, having an enveloped, single, positive-stranded RNA genome that encodes four major viral structural proteins, namely spike (S), envelope (E), membrane (M), and nucleocapsid (N) proteins 3–5, that follow the characteristic gene order [5′-replicase (*rep* gene), spike (S), envelope (E), membrane (M), nucleocapsid (N)-3′] with short untranslated regions at both termini (Figure 1). The viral membrane contains S, E, and M proteins, and the spike protein plays a vital functional role in viral entry. The *rep* gene encodes the non-structural protein and constitutes approximately two-thirds of the genome at the 5′ end. In detail, the S protein is in charge of receptor-binding and subsequent viral entry into host cells, and is therefore a major therapeutic target [26,27]. The M and E proteins play important roles in viral assembly, and the N protein is necessary for RNA synthesis.

The SARS-CoV genome has 29,727 nucleotides in length, including 11 open reading frames (ORFs). The SARS-CoV *rep* gene, containing about two-thirds of the genome, encodes at least two polyproteins (encoded by ORF1a and ORF1b) that undergo the process of cotranslational proteolysis. Between ORF1b and S of group 2 and some group 3 coronaviruses, there is a gene that encodes hemagglutinin-esterase [4], while this was not detected in SARS-CoV. This virus is significantly different from previously reported coronaviruses for many reasons, such as the short anchor of the S protein, the specific number and location of small ORFs, and the presence of only one copy of PLP^pro^.

The MERS-CoV genome is larger than that of SARS-CoV at 30,119 nucleotides in length, and comprises a 5′ terminal cap structure, along with a poly (A) tail at the 3′ end, as well as the *rep* gene containing 16 non-structural proteins (nsp1–16) at the 5′ end of the genome. Four structural proteins (S, E, M, and N) and five accessory proteins (ORF3, ORF4a, ORF4b, ORF5, and ORF8) constitute about 10 kb at the 3′ end of the genome. Unlike some other beta-coronaviruses, the MERS-CoV genome does not encode a hemagglutinin-esterase (HE) protein [1]. Genomic analysis of MERS-CoV implies the potential for genetic recombination during a MERS-CoV outbreak [9]. MERS-CoV and SARS-CoV possess five and eight accessory proteins, respectively, which might help the virus evade the immune system by being harmful to the innate immune response. These differences might lead to greater sensitivity to the effects of induction and signaling of type 1 interferons (IFNs) in MERS-CoV than SARS-CoV.

## 4. Comparative Pathology and Life Cycles of SARS-CoV and MERS-CoV

**Figure 2:**
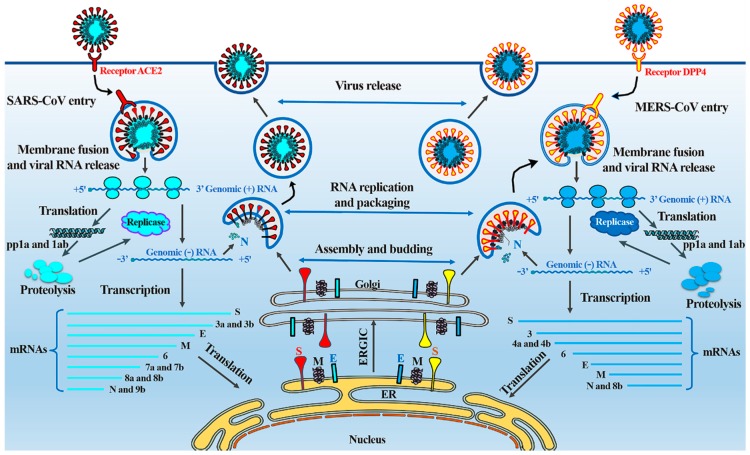
The life cycle of SARS-CoV and MERS-CoV in host cells. SARS-CoV and MERS-CoV enter target cells through an endosomal pathway. The S proteins of SARS and MERS bind to cellular receptor angiotensin-converting enzyme 2 (ACE2) and cellular receptor dipeptidyl peptidase 4 (DPP4), respectively. Following entry of the virus into the host cell, the viral RNA is unveiled in the cytoplasm. ORF1a and ORF1ab are translated to produce pp1a and pp1ab polyproteins, which are cleaved by the proteases that are encoded by ORF1a to yield 16 non-structural proteins that form the RNA replicase–transcriptase complex. This complex drives the production of negative-sense RNAs [(−) RNA] through both replication and transcription. During replication, full-length (−) RNA copies of the genome are produced and used as templates for full-length (+) RNA genomes. During transcription, a subset of 7–9 sub-genomic RNAs, including those encoding all structural proteins, is produced through discontinuous transcription. Although the different sub-genomic mRNAs may contain several open reading frames (ORFs), only the first ORF (that closest to the 5′ end) is translated. Viral nucleocapsids are assembled from genomic RNA and N protein in the cytoplasm, followed by budding into the lumen of the ERGIC (endoplasmic reticulum (ER)–Golgi intermediate compartment). Virions are then released from the infected cell through exocytosis. SARS-CoV, severe acute respiratory syndrome coronavirus; MERS-CoV, Middle East respiratory syndrome coronavirus; S, spike; E, envelope; M, membrane; N, nucleocapsid.

Both SARS and MERS cause severe pneumonia resulting from these novel coronaviruses, sharing some similarities in their pathogenesis (Figure 2) [28].

SARS is an emerging infectious viral disease characterized by severe clinical manifestations of the lower respiratory tract, resulting in diffuse alveolar damage. SARS-CoV spreads through respiratory secretions, such as droplets, via direct person-to-person contact. Upon exposure of the host to the virus, the virus binds to cells expressing the virus receptors, of which the angiotensin-converting enzyme 2 (ACE2) is one of the main receptors, and CD209L is an alternative receptor with a much lower affinity [29]. In the respiratory tract, ACE2 is widely expressed on the epithelial cells of alveoli, trachea, bronchi, bronchial serous glands [30], and alveolar monocytes and macrophages [31]. The virus enters and replicates in these target cells. The mature virions are then released from primary cells and infect new target cells [32]. Furthermore, as a surface molecule, ACE2 is also diffusely localized on the endothelial cells of arteries and veins, the mucosal cells of the intestines, tubular epithelial cells of the kidneys, epithelial cells of the renal tubules, and cerebral neurons and immune cells, providing a variety of susceptible cells to SARS-CoV [33,34]. Respiratory secretions, urine, stools, and sweat from patients with SARS contain infective viral particles, which may be excreted into and contaminate the environment. Atypical pneumonia with rapid respiratory deterioration and failure can be induced by SARS-CoV infection because of increased levels of activated proinflammatory chemokines and cytokines [35].

For MERS-CoV infection of humans, the primary receptor is a multifunctional cell surface protein, dipeptidyl peptidase 4 (DPP4, also known as CD26) [36], which is widely expressed on epithelial cells in the kidney, alveoli, small intestine, liver, and prostate, and on activated leukocytes [37]. Consistent with this, MERS-CoV can infect several human cell lines, including lower respiratory, kidney, intestinal, and liver cells, as well as histiocytes, as shown by a cell-line susceptibility study [38], indicating that the range of MERS-CoV tissue tropism in vitro was broader than that of any other CoV. MERS-CoV causes acute, highly lethal pneumonia and renal dysfunction with various clinical symptoms, including—but not restricted to—fever, cough, sore throat, myalgia, chest pain, diarrhea, vomiting, and abdominal pain [39,40]. Lung infection in the MERS animal model demonstrated infiltration of neutrophils and macrophages and alveolar edema [41]. The entry receptor (DPP4) for MERS-CoV is also highly expressed in the kidney, causing renal dysfunctions by either hypoxic damage or direct infection of the epithelia [42]. Remarkably, unlike SARS-CoV, MERS-CoV has the ability to infect human dendritic cells [43] and macrophages [44] in vitro, thus helping the virus to disrupt the immune system. T cells are another target for MERS-CoV because of their high amounts of CD26 [45]. This virus might deregulate antiviral T-cell responses due to the stimulation of T-cell apoptosis [45,46]. MERS-CoV might also lead to immune dysregulation [47] by stimulating attenuated innate immune responses, with delayed proinflammatory cytokine induction in vitro and in vivo [44,48,49].

## 5. SARS and MERS-CoV Spike Protein: A Key Target for Antivirals

### 5.1. Structure of the SARS-CoV and MERS-CoV Spike Protein

Trimers of the S protein make up the spikes of SARS-CoV and provide the formation of a 1255-amino-acids-length surface glycoprotein precursor. Most of the protein and the amino terminus are situated on the outside of the virus particle or the cell surface [50]. The expected structure of the S protein comprises four parts: a signal peptide located at the N terminus from amino acids 1 to 12, an extracellular domain from amino acids 13 to 1195, a transmembrane domain from amino acids 1196 to 1215, and an intracellular domain from amino acids 1216 to 1255. Proteases such as factor Xa, trypsin, and cathepsin L cleave the SARS-CoV S protein into two subunits, the S1 and S2 subunits. A minimal receptor-binding domain (RBD) located in the S1 subunit (amino acids 318–510) can combine with the host cell receptor, ACE2. The RBD displays a concave surface during interaction with the receptor. The entire receptor-binding loop, known as the receptor-binding motif (RBM) (amino acids 424–494), is located on the RBD and is responsible for complete contact with ACE2. Importantly, two residues in the RBM at positions 479 and 487 determine the progression of the SARS disease and the tropism of SARS-CoV [51,52]. Recent studies using civets, mice, and rats demonstrated that any change in these two residues might improve animal-to-human or human-to-human transmission and facilitate efficient cross-species infection [53]. The S2 subunit mediates the fusion between SARS-CoV and target cells, and includes the heptad repeat 1 (HR1) and HR2 domains, whose HR1 region is longer than the HR2 region.

Similar to SARS-CoV, during the infection process, the S protein of MERS-CoV is cleaved into a receptor-binding subunit S1 and a membrane-fusion subunit S2 [54,55,56,57]. The MERS-CoV S1 subunit also includes an RBD, mediating the attachment between virus and target cells [54,55,58,59]. Unlike SARS-CoV, MERS-CoV requires DPP4 (also known as CD26) as its cellular receptor [60,61] but not ACE2. The RBDs of MERS-CoV and SARS-CoV differ, although they share a high degree of structural similarity in their core subdomains, explaining the different critical receptors noted above [57,62]. The core subdomain of RBD is stabilized by three disulfide bonds, and includes a five-stranded antiparallel β-sheet and several connecting helices. The RBM comprises a four-stranded antiparallel β-sheet for connecting to the core via loops [57,62]. Two N-linked glycans, N410 and N487, are seated in the core and RBM, respectively. Particularly, the residues 484–567 of RBM take charge of interacting with the extracellular β-propeller domain of DPP4. The fusion core formation of MERS-CoV resembles that of SARS-CoV; however, it is different from that of other coronaviruses, such as the mouse hepatitis virus (MHV) and HCoV-NL63 [63,64,65,66].

### 5.2. Functions of the SARS-CoV and MERS-CoV S Protein

The SARS-CoV S protein plays pivotal roles in viral infection and pathogenesis [67,68]. The S1 subunit recognizes and binds to host receptors, and the subsequent conformational changes in the S2 subunit mediate fusion between the viral envelope and the host cell membrane [69,70]. The RBD in the S1 subunit is responsible for virus binding to host cell receptors [61,70,71]. ACE2 is a functional receptor for SARS-CoV that makes contact with 14 amino acids in the RBD of SARS-CoV among its 18 residues [53]. The RBD in the S1 subunit is responsible for virus binding to host cell receptors [61,70,71]. Position R453 in the RBD and position K341 in ACE2 play indispensable roles in complex formation [72]. Furthermore, the N479 and T487 in the RBD of the S protein are pivotal positions for the affinity with ACE2 [52], and R441 or D454 in the RBD influences the antigenic structure and binding activity between RBD and ACE2 [73]. From a pre-fusion structure to a post-fusion structure, binding of the RBD in the S1 subunit to the receptor ACE2 stimulates a conformational change in S2. Accordingly, the supposed fusion peptide (amino acids 770–788) [74] builds in the target cell membrane of the host. Meanwhile, a six-helix bundle fusion core structure is made up by the HR1 and HR2 domains for bringing the viral envelope and the target cell membrane into close proximity and contributing to fusion [74]. Resembling the S2 subunit of SARS-CoV, the MERS-CoV S2 subunit is in charge of membrane fusion. The HR1 and HR2 regions in S2 play essential and complementary roles [56,63]. Furthermore, SARS-CoV displays an alternative method of binding to the host cell via other potential receptors. Dendritic cell-specific intercellular adhesion molecule-3-grabbing non-integrin (DC-SIGN) and/or liver/lymph node-SIGN (L-SIGN) are two examples of such receptors [29,75]. Seven residue sites, at positions 109, 118, 119, 158, 227, 589, and 699 of the S protein displaying asparagine-linked glycosylation are crucial for DC-SIGN or L-SIGN-mediated virus entry. These residues, unlike those of the ACE2-binding domain, function independently of ACE2 [76].

### 5.3. Vaccines Based on the SARS-CoV and MERS-CoV S Protein

In order to control the outbreak of viruses, vaccinations were developed against SARS-CoV and MERS-CoV. There are various approaches of different vaccines, and the development and advantages/disadvantages of these are listed in Table 3 (this table includes updates about SARS-CoV and MERS-CoV since 2013; SARS-CoV-related parts were modified by Graham et al. in Nature Reviews Microbiology, 2013 [77]).

Importantly, among all the functional/non-functional structural proteins of SARS-CoV and MERS-CoV, the S protein is the principal antigenic component that induces antibodies to block virus-binding, stimulate host immune responses, fuse or neutralize antibodies and/or protect the immune system against virus infection. Therefore, the S protein has been selected as a significant target for the development of vaccines. It has been noted that antibodies raised against subunit S1 (amino acids 485–625) or S2 (amino acids 1029–1192) neutralize infection by SARS-CoV strains in Vero E6 cells [78,79]. Researchers have constructed an attenuated parainfluenza virus encoding the full-length S protein of the SARS-CoV Urbani strain for the vaccination of African green monkeys. This vaccine could protect monkeys from subsequent homologous SARS-CoV infection, demonstrating highly effective immunization with the S protein [80]. Other studies in a mouse model structured a DNA vaccine encoding the full-length S protein of the SARS-CoV Urbani strain that not only induced T-cell and neutralizing-antibody responses, but also stimulated protective immunity [81]. Furthermore, monkeys or mice were vaccinated with a highly attenuated, modified vaccine virus, Ankara, encoding the full-length S protein of the SARS-CoV strain HKU39849 or Urbani [82]. However, full-length S protein-based SARS vaccines may induce harmful immune responses, causing liver damage in the vaccinated animals or enhancing infection after being challenged with homologous SARS-CoV [83,84]. Researchers are thus concerned about the safety and ultimate protective efficacy of vaccines that include the full-length SARS-CoV S protein.

There are still no commercial vaccines available against MERS-CoV [26]. Multiple vaccine candidates targeting the S protein, which is responsible for viral entry, have been developed, including subunit vaccines [85,86], recombinant vector vaccines [87,88], and DNA vaccines [89,90]. Importantly, compared with other regions of the S protein, the RBD fragment induced the highest-titer IgG antibodies in mice [85]. Modified vaccines, including recombinant vectors of Ankara and adenoviruses expressing the MERS-CoV S glycoprotein, showed immunogenicity in mice [25]. Attenuated live vaccines also showed a protective function, but there were concerns regarding the degree of attenuation [91]. After intranasal vaccination with the CoV N protein, airway memory CD4 T cells were generated and mediated the protection following a CoV challenge [92]. These cells could induce anti-viral innate responses at an early stage of infection, and facilitated CD8 T-cell responses by stimulating recombinant dendritic cell migration and CD8 T-cell mobilization [92]. The stimulation of airway memory CD4 T cells should be regarded as an essential part of any HCoV vaccine strategy, because these CD4 T cells target a conserved epitope within the N protein that cross-reacts with several other CoVs [92]. Furthermore, DNA vaccines expressing the MERS-CoV S1 gene produced antigen-specific humoral and cellular immune responses in mice [89].

### 5.4. S Protein-Based Therapeutics for SARS-CoV and MERS-CoV

Despite the presence of extensive research reporting on SARS-CoV and MERS-CoV therapies, it was not possible to establish whether treatments benefited patients during their outbreak. In the absence of fundamental, clinically proven, effective antiviral therapy against SARS-CoV and MERS-CoV, patients mainly receive supportive care supplemented by diverse combinations of drugs. Several approaches are being considered to treat infections of SARS-CoV [113] and MERS-CoV (Table 4, MERS-CoV-related table previously reviewed by de Wit et al. in Nature Reviews Microbiology, 2016 [10]), including the use of antibodies, IFNs, and inhibitors of viral and host proteases.

The vital role of the S protein of SARS-CoV makes this protein an important therapeutic target, and numerous studies have explored potential therapeutics. Firstly, peptides that block RBD–ACE2-binding derived from both RBD [114] and ACE2 [76] could be developed as novel therapeutics against SARS-CoV infection. Secondly, peptides binding to the S protein interfere with the cleavage of S1 and S2. This step inhibits the production of functional S1 and S2 subunits and the consequent fusion of the viral envelope with the host cell membrane. Thirdly, anti-SARS-CoV peptides blocking the HR1–HR2 interaction by forming a fusion-active core have viral fusion inhibitory activity at the micromolar level [115,116,117]. However, the potential selection of escape mutants with altered host range phenotypes is one of the disadvantages of this strategy that needs further modification [118]. Furthermore, mouse monoclonal antibodies (mAbs) targeting assorted fragments of the SARS-CoV S protein have effectively inhibited SARS-CoV infection [79,119,120,121,122]. A series of neutralizing human mAbs were generated from the B cells of patients infected with SARS-CoV [123,124]. Another strategy used human immunoglobulin transgenic mice immunized with full-length SARS-CoV S proteins [125,126,127]. 80R and CR3014 binding to the ACE2 receptor are examples of S-specific mAbs [128,129].

Similarly, the therapeutic agents that have been developed against MERS-CoV are based on the S protein and basically restrain the binding of receptors or the fusion of membrane proteins, thereby leading to the inhibition of MERS-CoV infection. These methods mainly involve peptidic fusion inhibitors [56,63,116,130], anti-MERS-CoV neutralizing mAbs [86,131], anti-DPP4 mAbs [86,132,133], DPP4 antagonists [134], and protease inhibitors [135,136,137]. However, none of these anti-MERS-CoV curative agents are approved for commercial use in humans.

## 6. The Animal Models of SARS and MERS-CoV

International coordination and cooperation led to the rapid identification of SARS-CoV and MERS-CoV. Emergency control measures and laboratory detection systems which were put in place in response to SARS-CoV and MERS-CoV outbreaks were both exemplary. To establish optimal prevention and control strategies for SARS and MERS, numerous efforts to develop animal models were undertaken in several laboratories, despite the fact that some conflicting results have been reported. It is therefore necessary to compare and document the features and disadvantages of different animal models to better understand viral replication, transmission, pathogenesis, prevention, and treatment. Notably, several animal species were suggested as suitable disease models of SARS-CoV, but most laboratory animals are refractory or only semi-permissive to MERS-CoV infection.

### 6.1. Animal Models of SARS-CoV

SARS-CoV replication has been studied in mice, Syrian golden and Chinese hamsters, civet cats, and non-human primates. The most severe symptoms of SARS were observed in aged animals. To develop epidemiological symptoms that advanced age resulted in increased mortality, aged mouse model of SARS-CoV has been generated. Transgenic mice expressing human ACE2 were also developed to closely mimic SARS-CoV infection in humans. Some animal models have been tested and analyzed on the genomic and proteomics level to study the pathogenesis of SARS.

#### 6.1.1. Mouse Models

Mouse species that have been used as SARS-CoV-infected animal models include BALB/c [149,150], C57BL6 (B6) [151], and 129SvEv-lineage mice. The most relevant transgenic and knockout lines are accessible based on these susceptible animals [152]. Signal transducers and activators of transcription 1 (STAT1)-knockout and myeloid differentiation primary response 88 (MYD88)-knockout mice [149,151,153,154] are examples of mouse models with innate immune deficiency, and such animals display severe effects of the disease, such as pneumonitis, bronchiolitis, and weight loss, and often die within 9 days of infection. Notably, young mice require more mutations and passages than aged mice to produce SARS-CoV mouse-adapted strains. More severe pathological lesions and increased mortality were observed in one-year-old animals, along with fewer mutations at miscellaneous locations throughout the genome [98,155,156,157,158,159]. Intranasal inoculation of four- to eight-week-old BALB/c or B6 mice with SARS-CoV resulted in nasal turbinate in the upper respiratory tract and a high titer of virus replication in the lungs of the lower respiratory tract, and this model was highly reproducible without any signs of morbidity or mortality [149,151]. Neutralizing antibody responses could be generated in sub-lethally infected mice protecting recipients from subsequent lethal challenges, which probably reflected the situation in infected humans during an epidemic [160]. However, on day 2–3 post-infection (pi), virus replication in the respiratory tract peaked but was not accompanied by massive pulmonary inflammation or pneumonitis. By day 5–7 pi, the virus had been eliminated from the lungs [149,151]. It was obvious that viremia is common and long-lasting in patients, while it is rare and transient in mouse models [161]. Mice could therefore be used as a stable and reproducible animal model for the evaluation of vaccines, immune-prophylaxis, and antiviral drugs against SARS-CoV [81,96,109,124,149,162,163,164,165,166].

#### 6.1.2. Hamster Model

Golden Syrian and Chinese hamsters have also been evaluated and shown to be excellent models of SARS-CoV infection, owing to their high titer of virus replication in the respiratory tract, associated with diffuse alveolar damage, interstitial pneumonitis, and pulmonary consolidation [104,167,168,169]. On day 2 pi, peak levels of viral replication were detected in the lower respiratory tract, and the virus was cleared without obvious clinical illness 7–10 days after infection. Similarly to mice, infected hamsters also produced a protective neutralizing-antibody response to subsequent SARS-CoV challenges [104,170]. Resulting from the extremely high titers and reproducible pulmonary pathological lesions in SARS-CoV-infected hamsters, this animal model is ideal for studies on the immunoprophylaxis and treatment of SARS [104,170]. However, there are still limited resources in terms of genetically established animal lines and accurate immunological and cellular biomarkers for hamster models.

#### 6.1.3. Ferret Model

Ferrets were found to be susceptible to SARS-CoV infection [171] but could also transmit the virus at low levels by direct contact [84,172,173,174]. They showed diverse clinical symptoms in different studies [171,174]. Importantly, ferrets could develop fever, which is a characteristic clinical symptom of SARS-CoV-infected patients [93,175]. Similar to rodent models, infection of ferrets with SARS-CoV did not result in significant mortality. However, there are still some conflicting reports regarding the histopathological lesions and severity of clinical observations in the ferret model that require further investigation.

#### 6.1.4. Non-Human Primate Models

Several species of non-human primates (NHP) were evaluated as animal models for SARS. At least six NHP species were tested including three Old World monkeys: rhesus macaques [176,177,178,179,180], cynomolgus macaques [177,181,182], and African green monkeys [177]; and three New World monkeys: the common marmoset [183], squirrel monkeys, and mustached tamarins [176,177,178,181,182,183,184]. Except for squirrel monkeys and mustached tamarins [185], all of the evaluated NHP species facilitated the replication of SARS-CoV [186]. Virus replication was detected in the respiratory tract of rhesus macaques, cynomolgus macaques, and African green monkeys. Pneumonitis was observed in each of these species in different studies [176,177,178,182]. SARS-infected common marmosets displayed a fever, watery diarrhea, pneumonitis, and hepatitis [183]. Unfortunately, research into the clinical signs of disease in cynomolgus and rhesus macaques gave conflicting results and therefore needs further investigation. The main reason for the lack of reproducibility in such studies may be the limited sample size.

### 6.2. Animal Models of MERS-CoV

Small animal models of MERS infection are urgently needed to elucidate MERS pathogenesis and explore potential vaccines and antiviral drugs. Previous studies have demonstrated the difficulties in developing such a model, such as that mice [187,188], ferrets [134], guinea pigs [189], and hamsters [189] are not susceptible to experimental MERS-CoV infection mainly because their homologous DPP4 molecules do not function as receptors for MERS-CoV entry. After administering a high dose of MERS-CoV, no viral replication could be detected in these animals [190]. In an animal model using New Zealand white rabbits, regardless of the fact that detectable viral RNA existed in the respiratory tract and moderate necrosis was observed in nasal turbinates, the animals showed no clinical symptoms of disease [191]. In another study, attempts to infect hamsters with MERS-CoV were not successful [192]. However, despite this, MERS-CoV is a broad host-range virus in vitro [25], and there is hope that a reproducible and stable animal model for human MERS-CoV infection can be improved in the near future.

#### 6.2.1. Mouse Model for MERS Infection

Despite the fact that wild-type rodents are not susceptible to MERS-CoV infection [188], researchers have developed several models in which mice are susceptible to MERS-CoV infection [193,194,195]. The first mouse model of MERS infection reported in 2014 involved transducing animals with recombinant adenovirus 5 encoding human DPP4 (hDPP4) molecules intranasally, and this resulted in replication of MERS-CoV in the lungs. This mouse model also showed clinical symptoms of interstitial pneumonia, including inflammatory cell infiltration, and thickened alveolar and mild edema [195]. However, there are certain limitations to this model, such as the uncontrolled expression and distribution of hDPP4. In 2015, the establishment of hDPP4 transgenic mice was reported [194]. MERS-CoV could infect this mouse model effectively. However, similarly to SARS-CoV-infected ACE2 transgenic mice [196], systemic expressions led to multiple organ lesions [194], resulting in the death of the animals. Most recently, the homologous hDPP4 gene was used in several MERS transgenic mouse models [193,197]. Remarkably, hDPP4 knockin (KI) mice, where mouse DPP4 gene fragments had been displaced by homologous human DPP4 fragments, showed effective receptor binding. Furthermore, a mouse-adapted MERS-CoV strain (MERS_MA_) including 13–22 mutations was produced in the lungs of hDPP4-KI mice after 30 serial passages, causing effective weight loss and mortality in this mouse model [193]. Both this hDPP4-KI mouse and the MERS_MA_ strain provide better tools to explore the pathogenesis of MERS and potential novel treatments.

#### 6.2.2. Camelidae

As a reservoir of MERS-CoV, dromedary camels showed mild upper respiratory infections after the administration of MERS-CoV [198]. Oronasal infection of MERS-CoV in alpacas, a close relative within the Camelidae family, resulted in an asymptomatic infection with no signs of upper or lower respiratory tract disease [199,200]. Additionally, owing to their high cost and relatively large size, these animal models are not available for high-throughput studies of MERS.

#### 6.2.3. Non-Human Primates

NHPs, such as the rhesus macaques [201] and common marmosets [202], are useful models for studying the pathogenesis of mild MERS-CoV infection and evaluating novel therapies for humans, although the degree of replication and disease severity vary [192,201,203,204]. MERS-CoV caused transient lower respiratory tract infection in rhesus macaques, with associated pneumonia. Clinical signs were observed by day 1 pi and resolved as early as day 4 pi [201]. Relatively mild clinical symptoms were observed early on in infection without fatalities, indicating that rhesus macaques do not recapitulate the severe infections observed in human cases; however, treatment of MERS-CoV-infected rhesus macaques with IFN-α and ribavirin decreased virus replication, alleviated the host response, and improved the clinical outcome [205]. Infection of MERS-CoV in common marmosets demonstrated various extents of damage depending on the study, but successfully reproduced several features of MERS-CoV infection in humans. Importantly, one study indicated that the infection became progressive severe pneumonia [203], while other groups found that MERS-CoV-infected common marmosets only developed mild to moderate nonlethal respiratory diseases by intratracheal administration [206].

## 7. Role of Host Receptors in Animal Models of SARS-CoV and MERS-CoV

The reasons for host restriction, none or limited clinical symptoms observed in varies animal models are complexity. The interaction between the host receptor and functional proteins of SARS and MERS, respectively, plays an important and predominant role. In the context of animal models of SARS-CoV infection, researchers have compared the ACE2 amino acids that interact with the S protein RBD from several species. In agreement with the permissive nature of these species, the ACE2 residues of marmoset and hamster are similar to those of hACE2 [53]. By comparison, many residues of mouse ACE2 are different from those of hACE2, and this meets with decreased replication of SARS-CoV in mouse cells [207] and the lungs of young mice [149]. The changes at positions 353 (histidine) and 82 (asparagine) of rat ACE2 relative to hACE2 partially disrupt the S protein-DPP4 interaction and contribute to abrogation of binding. Interestingly, ferrets are permissive to SARS-CoV infection, but most of their ACE2 interaction residues are different from those of hACE2 [53], while many of the ACE2 residues between civet and ferret are the same, which may result in similar affinity [208]. For MERS-CoV, 14 residues of the S protein RBD have direct contact with 15 residues of hDPP4 [57]. Comparisons of human DPP4 binding affinity to that of other species indicated that human DPP4 had the highest affinity to the S protein of MERS-CoV, where the decreasing order of affinity is as follows: human > horses > camels > goats > bats [209]. Further evidence demonstrates that the host restriction of MERS-CoV remarkably depends on the sequence of DPP4, such as the characterization of amino acid residues at the connector of DPP4 with the RBD of S proteins in mice [187,210], hamsters, and cotton rats [210]. However, the multiplicity in severity of disease between rhesus macaques and common marmosets indicate that other host factors can perhaps affect the infection and replication of the virus, such as the presence of S-cleaving proteases [187]. In general, although the structural analysis of receptors-S protein interactions cannot fully explain all the observations for host restriction, they agree with the improved replication in several animal models and that it should be the premier and remarkable focus of small-animal model development. These special residues for host affinity are important to build up transgenic animal models enhancing the permissiveness and infection of SARS and MERS.

## 8. Outlook and Summary

Unlike SARS-CoV, which resolved without more reported cases, continued outbreaks of MERS-CoV present an ongoing threat to public health. It should be noted that no specific treatment is currently available for HCoVs, and further research into the pathogenesis of HCoV infection is therefore imperative to identify appropriate therapeutic targets. Accordingly, at present, the prevention of viral transmission is of utmost importance to limit the spread of MERS. The enormous ratio of nosocomial infections indicates that preventive measures in hospitals have not been sufficiently implemented. Additionally, as an emerging zoonotic virus, prevention of transmission from dromedary camels is another possibility to reduce the quantity of MERS cases. Regarding clinical therapies, a combination of treatment administered as early as possible and aimed at synchronously disrupting viral replication, inhibiting viral dissemination, and restraining the host response is likely to be most suitable, due to the acute clinical features of MERS with diffuse lung damage and the important role of immunopathology.

Potential treatments must undergo in vitro and in vivo studies to select the most promising options. The development of stable and reproducible animal models of MERS, especially in NHPs, is therefore a decisive step forward. The next step in the development of standardized and controllable therapies against SARS and MERS will be clinical trials in humans, validating a standard protocol for dosage and timing, and accruing data in real time during future outbreaks to monitor specific adverse effects and help inform treatment.

The comprehensive lessons and experiences that have resulted from the outbreaks of SARS and MERS provide valuable insight and advancements in how to react to future emerging and re-emerging infectious agents. Rapid identification of the pathogen via effective diagnostic assays is the first step, followed by the implementation of preventive measures, including raising awareness of the new agent, reporting and recording (suspected) cases, and infection control management in medical facilities. Studies are currently needed that focus on the epidemiology of these organisms, especially in terms of pathogen transmission and potential reservoirs and/or intermediate hosts. Animal models and prophylactic and therapeutic approaches should be promoted, followed by fast-tracked clinical trials.

Our increasing understanding of novel emerging coronaviruses will be accompanied by increasing opportunities for the reasonable design of therapeutics. Importantly, understanding this basic information will not only aid our public health preparedness against SARS-CoV and MERS-CoV, but also help prepare for novel coronaviruses that may emerge.

## Figures and Tables

**Table 1:** Epidemiology and biological characteristics of the severe acute respiratory syndrome coronavirus (SARS-CoV) and the Middle East respiratory syndrome coronavirus (MERS-CoV).

		SARS-CoV	MERS-CoV
**Genus**		Beta-CoVs, lineage B	Beta-CoVs, lineage C
**Possible Natural Reservoir**		Bat	Bat
**Possible Intermediary Host**		Palm civet	Dromedary camel
**Origin**		Guangdong province, China	Arabian Peninsula
**Clinical Epidemiology**	Total global number reported to WHO	More than 8098 people	2254 (from 2012 through 16 September 2018)
	Affected countries	29	27
	Number of deaths	916	800
	Mortality	More than 10%	More than 35%
	Transmission region	Globally	Regionally
	Transmission patterns	From animal to human; from human to human	
**The predominant receptor**		Human angiotensin-converting enzyme 2 (ACE2)	Human dipeptidyl peptidase 4 (DPP4 or CD26)
**Receptor** **distribution**		Arterial and venous endothelium; arterial smooth muscle; small intestine; respiratory tract epithelium; alveolar monocytes and macrophages	Respiratory tract epithelium; kidney; small intestine; liver and prostate; activated leukocytes
**Cell line susceptibility**		Respiratory tract; kidney; liver	Respiratory tract; intestinal tract; genitourinary tract; liver, kidney, neurons; monocyte; Tlymphocyte; and histiocytic cell lines
**Viral replication efficiency**		High	Higher
**Ability to inhibit IFN production**		Delayed recognition and proinflammatory response	Delayed recognition and proinflammatory response

**Table 2:** The genomic characterization of SARS-CoV and MERS-CoV.

		SARS-CoV	MERS-CoV
Length of nucleotides		29,727	30,119
Open reading frames (ORFs)		11	11
Structural protein		4	4
Spike protein (length of amino acids)		1255	1353
S1 subunit	Receptor-binding domain (RBD)	318–510	367–588
	Receptor-binding motif (RBM)	424–494	484–567
S2 subunit	Heptad repeat 1 (HR1) domains	892–1013	984–1104
	Heptad repeat 2 (HR2) domains	1145–1195	1246–1295
Non-structural proteins (NSPs)		At least 5	16
Accessory proteins		8	5
A characteristic gene order		5′-replicase ORF1ab, spike (S), envelope (E), membrane (M), and nucleocapsid (N)-3′	

**Table 3:** Vaccine strategies of SARS-CoV and MERS-CoV.

Vaccine Strategy	Process of Production	References		Advantages	Disadvantages
		SARS	MERS		
Inactivated virus vaccines	Virus particles are inactivated by heat, chemicals, or radiation	Whole virus, with or without adjuvant (promote an effective immune response against the inactivated pathogen) [93,94]	Whole virus, with or without adjuvant (promote an effective immune response against the inactivated pathogen) [91,95]	Maintained virus particles structure; rapidly develop; easy to prepare; safety; high-titer neutralizing antibodies [93]; protection with adjuvant [96,97].	Potential inappropriate for highly immunosuppressed individuals; possible T_H_2 cell-distortive immune response [98,99].
Live-attenuated virus vaccines	Attenuated the virulence, but still keeping it viable by mutagenesis or targeted deletions	Envelope protein deletion [100]; non-structural protein 14 (nsp14) and exonuclease (ExoN) inactivation [101]	Full-length infectious cDNA clone or mutant viruses [102]	Inexpensive; quick immunity; less adverse effect; activates all phases of the immune system [103]; more durable immunity; more targeted [77].	Phenotypic or genotypic reversion possible; need sufficient viral replication [77].
Viral vector vaccines	Genetically engineered unrelated viral genome with deficient packaging elements for encoding targeted gene	Spike and nucleocapsid proteins [100,104]	Spike and nucleocapsid proteins [87,88]	Safety; stronger and specific cellular and humoral immune responses [77].	Varies inoculation routes may produce different immune responses [96]; possibly incomplete protection; may fail in aged vaccinees; possible T_H_2 cell-distortive immune response [105].
Subunit vaccines	Antigenic components inducing the immune system without introducing viral particles, whole or otherwise.	Spike and nucleocapsid proteins [53,59,106]	Spike and nucleocapsid proteins [85,86,107,108]	High safety; consistent production; can induce cellular and humoral immune responses; high-titer neutralizing antibodies [109].	Uncertain cost-effectiveness; relatively lower immunogenicity; need appropriate adjuvants [77].
DNA vaccines	Genetically engineered DNA for directly producing an antigen	Spike and nucleocapsid proteins [110,111]	Spike and nucleocapsid proteins [89,90]	Easier to design; high safety; high-titer neutralizing antibodies [110].	Lower immune responses; potential T_H_2 cell-distortive immune response results; potential ineffective; possibly delayed-type hypersensitivity [112].

**Table 4:** Potential therapeutics for severe acute respiratory syndrome (SARS) and MERS.

Treatment	Stage of Development	
	SARS (Notes)	MERS (Notes)
Host protease inhibitors	Effective in mouse models [138]	In vitro inhibition [138]
Viral protease inhibitors	In vitro inhibition [139]	In vitro inhibition [140]
Monoclonal and polyclonal antibodies	Effective in mouse, ferrets, golden Syrian hamster [124,141,142] and non-human primate models [143,144]	Effective in mouse, rabbit, and non-human primate models [10,145]
Convalescent plasma	Off-label use in patients [146,147]	Effective in a mouse model; clinical trial approved [10]
Interferons	Off-label use in patients (often in combination with immunoglobulins or thymosins) [146,147]	Effective in non-human primate models; off-label use in patients (often in combination with a broad-spectrum antibiotic and oxygen) [10]
Ribavirin	Off-label use in patients (often in combination with corticosteroids) [146,147]	Effective in a non-human primate model; off-label use in patients (often in combination with a broad-spectrum antibiotic and oxygen) [10]
Lopinavir and ritonavir	Off-label use in patients (improved the outcome in combination with ribavirin) [146,147]	Effective in a non-human primate model; off-label use in patients [10,148]
**Common Feature**	None of these therapeutic agents are approved for commercial use in humans	
